# A Case of Nonanatomic Alignment: Acute Radial Head Dislocation Following Radial Shaft Osteosynthesis

**DOI:** 10.1155/cro/3498572

**Published:** 2026-07-18

**Authors:** Khalid H. Almaazmi, Saif S. Aldhuhoori

**Affiliations:** ^1^ Department of Orthopedic Surgery, Sheikh Shakbout Medical City, Abu Dhabi, UAE; ^2^ Department of Orthopedic Surgery, Zayed Military Hospital, Abu Dhabi, UAE, zmh-elibrary.com

**Keywords:** both bone forearm fractures, fracture malreduction, internal fracture fixation, radial bow, radial head dislocation, radius fractures, ulna fractures

## Abstract

Radial head dislocation after both bone forearm fractures (BBFF) fixation is rare. The management of BBFF involves open reduction and internal fixation (ORIF) to restore anatomy and function. Anatomic reduction of the radius and ulna is required to restore the unique geometric relationship of the forearm and allow normal rotation. Nonanatomic reduction of the radius can result in postoperative radial head dislocation, disrupting forearm function and stability.

## 1. Introduction

Radius and ulnar shaft fractures, also known as both bone forearm fractures (BBFF), are uncommon in adults when compared with distal radius fractures [1]. Radial shaft fracture associated with radial head dislocation is a rare injury pattern and, in most published reports, the dislocation is already present at the time of trauma [2–5]. Classic Monteggia fracture‐dislocation refers to a proximal ulnar fracture associated with radial head dislocation [2, 6], whereas related patterns involving disruption of the proximal radioulnar joint have been described in the literature [7–9]. In the present case, however, the initial radiographs did not demonstrate radial head dislocation; instead, the radial head dislocated only after osteosynthesis. As such, this case report is a case of acute radial head dislocation post‐ORIF of BBFF. CARE guidelines were utilized to report this case report.

## 2. Case Report

A 38‐year‐old, healthy, right‐hand–dominant male sustained a fall down a flight of stairs at his home, presenting to the emergency department with acute forearm pain, swelling, and deformity. There were no associated injuries.

Physical examination revealed a puncture wound along the ulnar aspect of the distal forearm. It also revealed deformity and swelling of the forearm. The neurovascular examination was unremarkable, with palpable distal pulses, brisk capillary refill, and soft forearm compartments.

**Figure 1:**
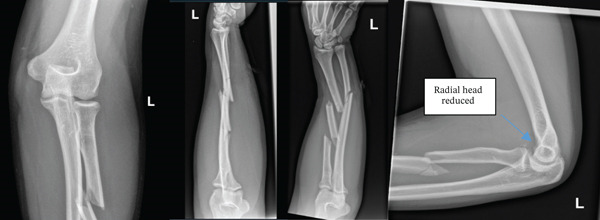
Oblique ulnar fracture and segmental radial shaft fracture.

**Figure 2:**
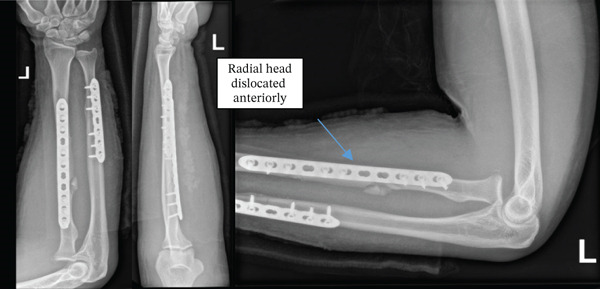
AP and lateral radiographs after application of the backslab.

Plain radiographs were obtained to further evaluate the injury. Anteroposterior (AP) and lateral views of the forearm showed radius and ulnar shaft fractures or BBFF. The fractures were oblique ulnar shaft and segmental radial shaft fractures (Figure 1). There was no dislocation of the radial head. Tetanus prophylaxis and antibiotics were administered. Irrigation of the puncture wound and application of an above elbow back slab was done (Figure 2).

## 3. Operative Management: First Procedure

**Figure 3:**
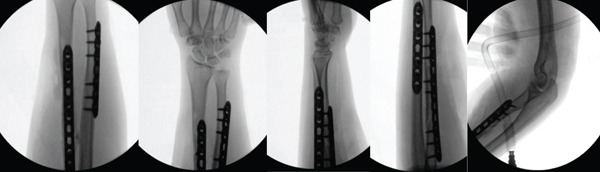
Intraoperative fluoroscopic images.

**Figure 4:**
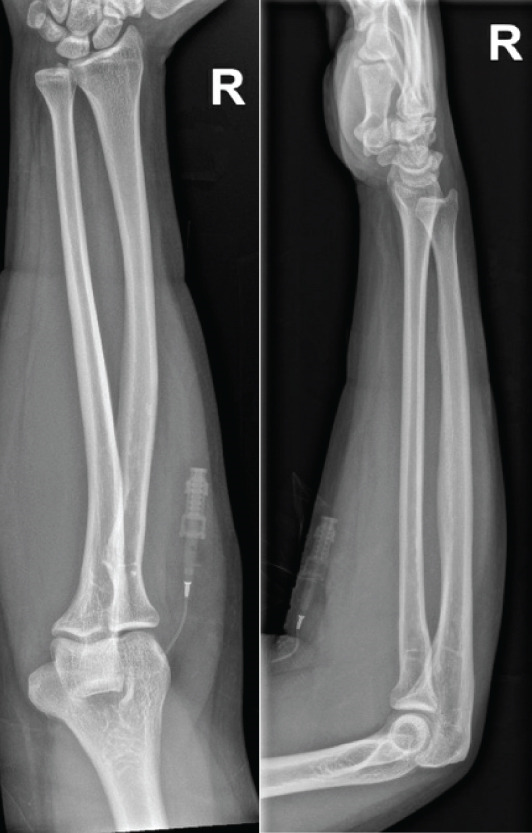
Postoperative radiographs demonstrating anterior radial head dislocation.

The patient was taken to the operating room. Positioned supine, he underwent debridement of the puncture wound. The ulna fracture was addressed first and stabilized with a 3.5 mm 7‐hole LC‐DCP plate (Synthes) using three cortical nonlocking screws proximally and three distally, followed by layered closure with Vicryl and Prolene sutures. A Henry approach was then used for the segmental radial shaft fracture, as it provides adequate exposure of the radial shaft, allows direct visualization of the fracture site, protects the posterior interosseous nerve, and minimizes unnecessary soft‐tissue disruption. Temporary stabilization was achieved with Kirschner wires, and definitive fixation was performed with a 3.5 mm 11‐hole LC‐DCP plate (Synthes). The screw configuration included three screws proximally, three distally, and two screws in the segmental fragment (Figure 3). In retrospect, the plate was not sufficiently contoured to reproduce the native radial bow. Given the segmental fracture configuration, this likely reflected an intraoperative assessment error regarding restoration of radial alignment and bow rather than a deliberate acceptance of a nonanatomic construct. Immediate postoperative radiographs subsequently revealed anterior dislocation of the radial head (Figure 4).

## 4. Operative Management: Revision Procedure

**Figure 5:**
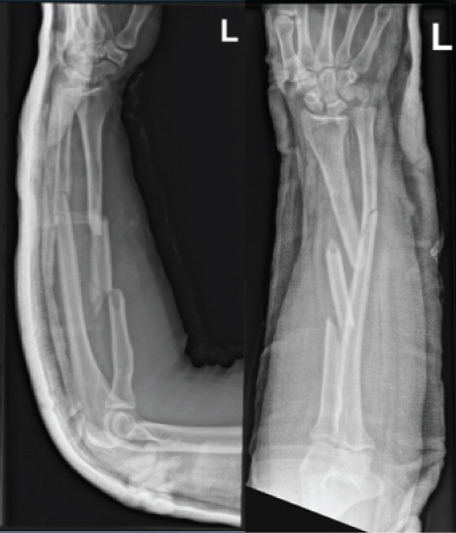
Forearm radiograph of the contralateral side.

The patient returned to the operating room the following day. Before revision, a contralateral forearm radiograph was obtained to better assess the native radial bow and guide implant contouring (Figure 5).

**Figure 6:**
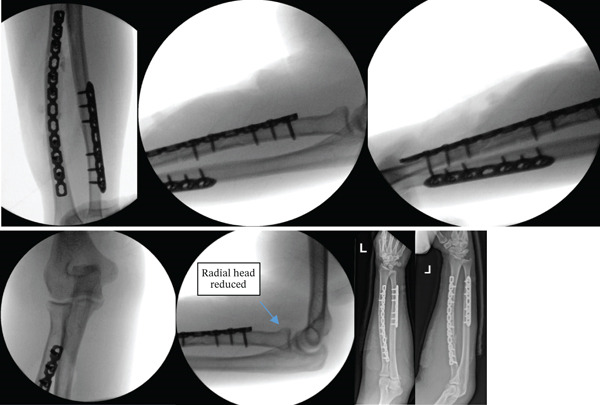
Intraoperative fluoroscopy after revision demonstrating restoration of radial bow and reduction of the radial head.

A 3.5 mm reconstruction plate was then contoured to mimic the native bow and fixed with screws proximally and distally. Intraoperatively, elbow and distal radioulnar joint stability were assessed through full ranges of flexion, extension, pronation, and supination. A varus stress test was also performed after revision and demonstrated a stable elbow without recurrent radial head subluxation or dislocation. Intraoperative fluoroscopy confirmed satisfactory plate position, restoration of the radial bow, and concentric reduction of the radial head (Figure 6).

## 5. Results

Two weeks postoperatively, sugical wounds healed and neurovascular examination is intact. Physiotherapy started in Week 3 with passive range of motion (ROM) elbow, forearm, wrist and hand then followed by active ROM. At 3 months follow‐up, the patient had full ROM of pronation and supination of the forearm and flexion and extension of the elbow. He had no neurovascular deficit.

## 6. Discussion

This case highlights a rare but important complication of BBFF fixation: acute postoperative radial head dislocation following nonanatomic reconstruction of the radius. Unlike classic Monteggia injuries, the radial head was aligned on initial radiographs and the dislocation became apparent only after fixation, making this a distinct postoperative problem rather than a primary fracture‐dislocation pattern. Furthermore, the intraoperative x‐rays confirmed reduced radial head with no dislocation.

### 6.1. The Importance of Radial Bow Restoration

The forearm functions as a highly coordinated osseoligamentous unit, and restoration of radial length, alignment, rotation, and bow is essential for normal pronation and supination [10]. Schemitsch and Richards demonstrated that restoration of the normal amount and location of the radial bow is associated with improved forearm rotation and function [11]. In their radiographic method, the point of maximum radial bow is typically located at approximately 60% of the radial length measured from the bicipital tuberosity to the ulnar side of the distal radius, with a mean magnitude of about 15 mm [11]. These data support the concept that even subtle nonanatomic reconstruction can alter forearm kinematics and compromise radiocapitellar stability.

**Figure 7:**
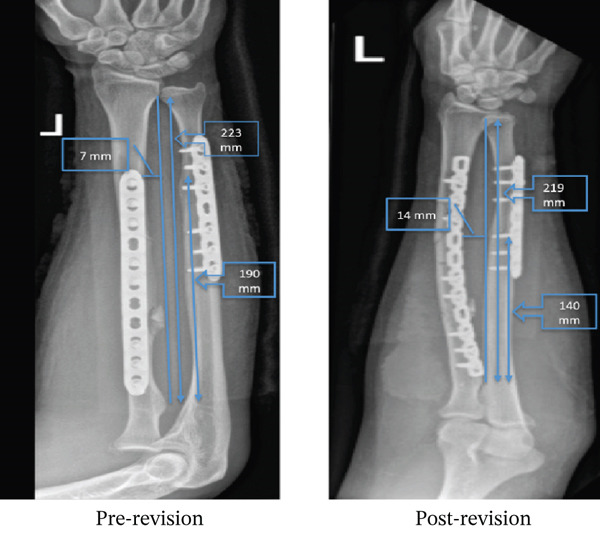
Prerevision and postrevision radiographs and measurements of maximum radial bow and location of maximum radial bow.

The maximum radial bow and location of maximum radial bow are quantified based on standard AP view of the forearm. The maximum radial bow is measured by drawing a line from the bicipital tuberosity to the radius ulnar notch. Another perpendicular line is drawn from this line to the radial cortex representing the maximum radial bow that typically ranges between 10–15 mm. The location of maximum radial bow is presented as a percentage by measuring the distance from the bicipital tuberosity to the previously made perpendicular line (point of maximum bow) divided by the total radial bow (distance from the bicipital tuberosity to the radius ulnar notch). The location of maximum radial bow ranges 50%–65% of radial length. Our case prerevision and postrevision measurements for maximum radial bow and location of maximum radial bow are shown in Figure 7.

### 6.2. Proposed Mechanisms for Post‐ORIF Radial Head Dislocation

Several mechanisms may explain postoperative radial head dislocation in this setting. First, the radial head may have transiently dislocated at the time of injury and spontaneously reduced before the preoperative radiographs, leaving behind residual soft‐tissue insufficiency. Second, nonanatomical fixation of the radius may alter the forearm axis sufficiently to unmask instability at the proximal radioulnar and radiocapitellar joints [12, 13]. Third, injuries involving the proximal third of the radial shaft, especially when associated with a butterfly fragment, may include soft‐tissue attachments from the interosseous membrane and thereby contribute to proximal forearm instability [7–9]. These possibilities are consistent with prior reports of delayed radial head dislocation after radial shaft fracture fixation and with published descriptions of ipsilateral radial shaft fracture‐radial head dislocation patterns.

### 6.3. Surgical Techniques and Considerations

In the present case, the most likely explanation for the initial failure was inadequate restoration of the native radial bow. This was not an intentional compromise, but more likely an intraoperative assessment error related to the segmental nature of the radial shaft fracture, which made appreciation of the final three‐dimensional alignment more difficult. Revision with a contoured reconstruction plate, guided by the contralateral forearm radiograph, restored the radial bow and allowed concentric reduction of the radial head. Intraoperative assessment should include not only fluoroscopic confirmation of fracture alignment, but also evaluation of the radiocapitellar relationship, forearm rotation, distal radioulnar joint stability, and elbow stability. In our case, after revision fixation, full pronation‐supination assessment and varus stress testing confirmed a stable elbow.

There are some strategies to be considered when a long anatomic metadiaphyseal plate is not available. Two overlapping plates or contouring a straight plate techniques can be considered for fixation of segmental radial shaft fractures. When two overlapping plates are used, the distal plate should be thinner and can act as a buttress plate; the shaft fracture is fixed with a thicker plate that acts as a compression or bridging plate.

The long straight plate can be contoured intraoperatively or preoperatively using a forearm skeletal model. Because the radius is wide distally, the plate can be translated slightly radially in the distal aspect so that the proximal aspect of the plate overlays the bone and not the interosseous membrane. Another trick is not to fully tighten the screws that are placed at each end of the plate. Once the fracture is anatomically reduced, the screws are tightened. The plate on the proximal radius should be placed radial to the bicipital tuberosity to avoid biceps tendon impingement at its insertion on the tuberosity, plus bicortical screws will be placed in the center of the proximal radius.

## 7. Conclusion

This case demonstrates that acute radial head dislocation may occur after BBFF fixation when the radius is not reconstructed anatomically. Restoration of radial bow, length, alignment, and rotation is fundamental to maintaining proximal forearm stability. When fracture morphology is complex, careful intraoperative assessment of the radiocapitellar joint, forearm rotation, and distal radioulnar joint stability is essential, and comparison with the contralateral forearm may be helpful.

## Funding

No funding was received for this manuscript.

## Ethics Statement

Patient consent was obtained.

## Conflicts of Interest

The authors declare no conflicts of interest.

## Data Availability

The data that support the findings of this study are available from the corresponding author upon reasonable request.

## References

[1] Jónsson B. , Bengnér U. , Redlund-Johnell I. , and Johnell O. , Forearm Fractures in Malmo, Sweden. Changes in the Incidence Occurring During the 1950s, 1980s and 1990s, Acta Orthopaedica Scandinavica. (1999) 70, no. 2, 129–132, 10.3109/17453679909011249, 10366911. 10366911

[2] Haddad F. S. , Manktelow A. R. J. , and Dorrell J. H. , Radial Head Dislocation With Radial Shaft Fracture, Injury. (1995) 26, no. 7, 502–503, 10.1016/0020-1383(95)00077-M. 7493795

[3] Adhikari A. , Acharya S. , and Bhandari R. , Radial Head Dislocation With Ipsilateral Proximal Shaft of Radius Fracture: A Case Report, Journal of Nepal Medical Association. (2020) 58, no. 226, 416–418, 10.31729/jnma.4991. PMC7580340 32788759

[4] Ahn E. H. , Jung I. H. , Oh J. H. , and Shin K. C. , Ipsilateral Radial Head Dislocation and Radial Shaft Fracture: A Case Report, Journal of the Korean Orthopaedic Association. (1992) 27, no. 3, 10.4055/jkoa.1992.27.3.844.

[5] Mehara A. K. and Bhan S. , Ipsilateral Radial Head Dislocation With Radial Shaft Fracture, Journal of Trauma and Injury. (1993) 35, 958–959. 10.1097/00005373-199312000-00027 8264000

[6] Moss J. P. and Bynum D. K. , Diaphyseal Fractures of the Radius and Ulna in Adults, Hand Clinics. (2007) 23, no. 2, 143–151, 10.1016/j.hcl.2007.03.002. 17548006

[7] Linzel D. , Ring D. , and Jupiter J. , Diaphyseal Fracture of the Radius With Dislocation of the Proximal Radioulnar Joint, Journal of Trauma and Injury. (2008) 64, 503–506, 10.1097/TA.0b013e31802e70ea. 18301222

[8] Shamian B. and Capo J. T. , Isolated Radial Shaft Fracture With Unreducable Posterior Dislocation of the Radial Head and Rupture of the Lateral Collateral Ligament: A Case Report, Journal of Clinical Orthopaedics and Trauma. (2012) 3, no. 2, 126–129, 10.1016/j.jcot.2012.04.005, 26403453. 26403453 PMC3872807

[9] Singh J. , Kalia A. , and Dahuja A. , Ipsilateral Radial Head Dislocation and Proximal One-Third Radial Shaft Fracture in an Adult: A Case Report, Open Orthopaedics Journal. (2018) 12, no. 1, 189–195, 10.2174/1874325001812010189, 29997706. 29997706 PMC5997876

[10] Richard M. J. , Ruch D. S. , and Aldridge J. M. , Malunions and Nonunions of the Forearm, Hand Clinics. (2007) 23, no. 2, 235–243, 10.1016/j.hcl.2007.02.005. 17548014

[11] Schemitsch E. H. and Richards R. R. , The Effect of Malunion on Functional Outcome After Plate Fixation of Fractures of Both Bones of the Forearm in Adults, Journal of Bone and Joint Surgery. (1992) 74, no. 7, 1068–1078, 10.2106/00004623-199274070-00014, 1522093. 1522093

[12] Yamazaki H. , Kato H. , Yasutomi T. , Murakami N. , and Hata Y. , Delayed Radial Head Dislocation Associated With Malunion of Radial Shaft Fracture: A Case Report, Journal of Shoulder and Elbow Surgery. (2007) 16, no. 2, e18–e21, 10.1016/j.jse.2006.05.014, 17399620. 17399620

[13] Yang J. , Zhang J. , and Yang Z. , Delayed Radial Head Dislocation After Radial Shaft Fracture Fixation: A Case Report and Review of the Literature, BMC Surgery. (2022) 22, no. 1, 10.1186/s12893-022-01514-1. PMC8851837 35172793

